# Environmental tobacco smoke is a major contributor to lead, cadmium, and arsenic in settled house dust

**DOI:** 10.1016/j.chemosphere.2025.144820

**Published:** 2026-01-02

**Authors:** Nicolas Lopez-Galvez, E. Melinda Mahabee-Gittens, Penelope J.E. Quintana, Ashley L. Merianos, Nathan G. Dodder, Eunha Hoh, Lara Stone, Kayo Watanabe, Georg E. Matt

**Affiliations:** a San Diego State University, School of Public Health, 5500 Campanile Drive, San Diego, CA, 92182-4611, USA; b Cincinnati Children's Hospital Medical Center, Division of Emergency Medicine, Department of Pediatrics, University of Cincinnati, College of Medicine, 3333 Burnet Avenue, Cincinnati, OH, 45229, USA; c San Diego State University, Department of Psychology, 5500 Campanile Drive, San Diego, CA, 92182-4611, USA; d University of Cincinnati, School of Human Services, PO Box 210068, Cincinnati, OH, 45221-0068, USA

## Abstract

Tobacco smoke contains toxic chemicals, including heavy metals lead (Pb), cadmium (Cd), and arsenic (As), that are found in secondhand and thirdhand smoke, the chemical residue that lingers and accumulates in indoor environments. This study examined tobacco smoke's contribution to heavy metal accumulation in house dust after smoking stops. Dust samples were collected from 179 homes of cigarette smokers with young children and analyzed for nicotine and tobacco-specific nitrosamines (TSNAs) using isotope-dilution liquid chromatography-tandem mass spectrometry. Pb, Cd, and As were analyzed by inductively coupled plasma-mass spectrometry. Bivariate correlation and multivariable models were used to examine associations between tobacco use markers and heavy metal dust loading, controlling for sociodemographic, home, and smoking characteristics. All samples contained Pb (geometric mean: 34.2 μg/m^2^), Cd (0.40 μg/m^2^), As (1.6 μg/m^2^), nicotine (15.7 μg/m^2^), and TSNAs (6.0 ng/m^2^); 100 % exceeded the current EPA standard for Pb (0 μg/ft^2^). Metals correlated positively with nicotine (Pb: r = 0.68; Cd: r = 0.70; As: r = 0.70; all p < 0.001) and TSNAs (Pb: r = 0.50; Cd: r = 0.47; As: r = 0.51; all p < 0.001). Nicotine loading was strongly associated with higher loading of the other contaminants (Pb: $\hat{\beta }=0.68$; Cd: $\hat{\beta }=0.71$; As: $\hat{\beta }=0.69$; TSNAs: $\hat{\beta }=0.83$; all p < 0.001). The semi-partial R^2^ values showed that nicotine uniquely explained 43 % of Pb, 58 % of Cd, and 54 % of As dust-loading variance. Our model projected that, in a fully tobacco-free home, dust loadings of Pb, Cd, and As would decrease by 87 %, 49 %, and 38 %, respectively. This study provides compelling evidence that tobacco smoke residue is a significant yet underrecognized source of toxic metals in household dust, independent of established factors such as housing age. It substantially contributes to indoor lead, cadmium, and arsenic levels. Given children's heightened vulnerability, these findings highlight the urgency of smoke-free policies and thirdhand smoke remediation.

## GRAPHICAL ABSTRACT

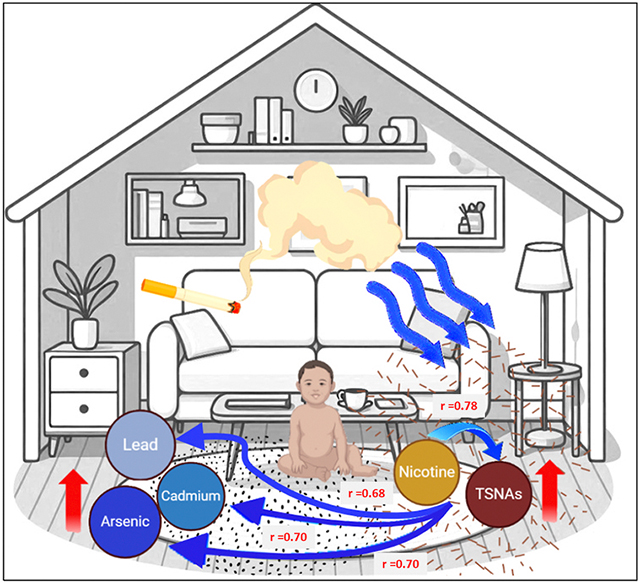

## 1. Introduction

Tobacco smoke contains thousands of harmful substances, many of which pose significant risks to human health and safety. Toxic metals and metalloids such as lead (Pb), cadmium (Cd), and arsenic (As) have been identified and measured in tobacco and tobacco smoke (Chiba and Masironi, 1992). Hence, smoking contributes to non-smokers’ intake of these contaminants through secondhand smoke (SHS) exposure. SHS exposure has been associated with elevated Pb and Cd levels in the blood of both children and adults (Rahmani et al., 2024). Further, SHS has been associated with increased levels of Pb in children's saliva (Gatzke-Kopp et al., 2023). While SHS dissipates relatively quickly, it can adhere to surfaces and accumulate in household dust, resulting in a persistent toxic residue referred to as thirdhand smoke (THS) (Jacob et al., 2017). Unlike SHS, as THS ages, it undergoes dynamic chemical transformations that can generate new toxicants and can remain embedded in materials for weeks or months, creating a prolonged source of exposure (Wang, Y., & Gu, J., 2025). For instance, when THS forms over time, primary tobacco smoke constituents like nicotine can react with various indoor compounds such as ozone or nitrous acid, a common pollutant generated by gas stoves and heaters, producing new carcinogenic compounds (Sleiman et al., 2010).

Many harmful pollutants in THS, including nicotine, tobacco-specific nitrosamines (TSNAs), and polycyclic aromatic hydrocarbons, can accumulate and persist in carpets, furniture, upholstery, building materials, and other objects for years, even after SHS has cleared (Matt et al., 2010, 2021, 2021b). This is concerning because THS residues can be re-emitted and resuspended into the environment, where they may be subsequently inhaled, absorbed through the skin, or ingested, leading to involuntary prolong exposure among residents, especially vulnerable populations such as children (Matt et al., 2021, 2021b).

Although the presence of toxic metals in tobacco smoke is well documented, the quantification of trace metals in THS remains scarce, representing a critical gap with direct implications for public health (Matt et al., 2021, 2021b; Tang et al., 2024). The detection of Pb, Cd, and As in THS is of critical importance, given their well-established toxicity and potential to induce serious adverse health outcomes. Prior research indicates that exposure to Pb, Cd, and As is associated with impairments in children's verbal and non-verbal cognition, behavior, and motor skills (Sanders et al., 2015). Cd exposure has also been linked to kidney damage, lung diseases, and an increased risk of cancer (Satarug et al., 2010). In addition, arsenic is classified as a human carcinogen by the International Agency for Research on Cancer (IARC). Exposure to arsenic has been associated with an increased risk of skin, lung, bladder, and kidney cancers, as well as cardiovascular diseases (Abdul et al., 2015). Prolonged exposure to Pb in children can lead to lasting cognitive impairments and affect their overall development (Naranjo et al., 2020).

In response to mounting evidence on the health risks of lead-contaminated environments, the U.S. Environmental Protection Agency (EPA) has progressively strengthened its regulatory standards for residential lead exposure. In 2019, the EPA revised its Dust-Lead Hazard Standards (DLHS), lowering the permissible lead loading on residential floors from 40 μg/ft^2^ to 10 μg/ft^2^ (Shi and Wang, 2021). Building on this momentum, a landmark update finalized in 2024 redefined the DLHS as the Dust-Lead Reportable Level (DLRL), now set at any detectable level of lead in dust, determined by the lowest concentration reliably measurable. This rule is currently in effect and marks a significant shift toward more precautionary thresholds (EPA, 2024). Concurrently, the EPA revised its Dust-Lead Clearance Levels (DLCL) now termed Dust-Lead Action Levels (DLAL) for contaminated homes to 5 μg/ft^2^ for floors. These updates reflect a growing scientific and policy consensus that no level of lead exposure is safe for children. Internationally, the World Health Organization (WHO) echoes this position, calling on governments to eliminate Pb sources wherever feasible (WHO, 2021). Exposure to these pollutants in household dust primarily occurs through incidental ingestion, inhalation, and dermal contact pathways (Ollson et al., 2017). Young children and toddlers face an increased vulnerability to these pollutants, as they are active close to the floor, frequently put their hands and objects into their mouths, and are at a sensitive developmental stage (Roberts et al., 2009). The levels of heavy metals found in indoor dust are generally influenced by a combination of external (e.g., nearby industrial activities, vehicle emissions) and internal factors (e.g., building materials, paint, house age, ventilation, floor covering, and cleaning frequency) (Shi and Wang, 2021). Limited investigations have been conducted on the contribution of tobacco smoke to house dust metal contamination, with some studies finding that smoking in the home explained some of the variability in lead loading; however, the contribution from smoking was not a focus of these studies (Gaitens et al., 2009; Lucas et al., 2014). Although nicotine in house dust has been considered a reliable exposure marker of THS, only a few studies have investigated the association of nicotine in house dust with metals (Matt et al., 2021, 2021b; Willers et al., 2005). Dust nicotine concentration was not significantly associated with Pb and Cd concentrations in a study of 23 asthmatic children in Sweden (Willers et al., 2005). In contrast, a recent study in California, USA, among 60 multiunit homes with indoor smoking bans, determined that Pb and Cd are important constituents of THS, as the loading levels of these pollutants in dust were significantly associated with the dust loading of nicotine (Matt et al., 2021, 2021b). These conflicting results likely reflect differences in sample size, population characteristics, and exposure variability. Together, they underscore the need for further research on the co-occurrence of nicotine and toxic metals in settled dust.

Therefore, the objective of this study was to evaluate nicotine in household dust as a marker of thirdhand smoke and examine its association with toxic metals, lead, cadmium, and arsenic, after adjusting for known contributors to indoor metal levels, including housing characteristics (e.g., age, structure, size), occupant behaviors and socioeconomic factors. To our knowledge, this is the first study to investigate these associations in this context, aiming to determine whether tobacco smoke represents an underrecognized and ongoing source of indoor metal exposure.

## 2. Methods

Participants were recruited from a large, randomized controlled trial of a parental tobacco cessation intervention, the “Healthy Families” study (R01HD083554) in Cincinnati, Ohio, U.S. (N = 750; see details elsewhere) (Mahabee-Gittens et al., 2017, 2021). A subsample of 219 children (ages 0–11 years old) of active smokers was further evaluated, and environmental samples were collected from their homes. Before participating, all individuals provided written informed consent that was approved by the Institutional Review Board (approval number 2017–5157) of Cincinnati Children's Hospital. Of the 219 participants included in the clinical trial study, N = 179 provided household dust samples to be analyzed for nicotine, Pb, Cd, As, and TSNAs.

### 2.1. Questionnaire

To gather sociodemographic, smoking behavior, and housing information, all participants completed an electronic questionnaire (details presented elsewhere) (Mahabee-Gittens et al., 2017). Collected information included children's sociodemographic characteristics (e.g., parent education, annual household income), housing type (single--family home, apartment building, multiunit home such as townhome), home age, number of cigarette smokers in a household, and home smoking bans.

### 2.2. Dust sampling and instrumental analysis of nicotine, lead, cadmium, arsenic, and TSNAs

We used a high-volume-small surface-sampler cyclone vacuum (HVS4, CS3, Venice, Florida, USA) to collect a dust sample from a 1 m^2^ area (or from a larger area if needed). All dust samples were stored at ≤ −20 °C until analysis at the Environmental Health Laboratory at San Diego State University. Nicotine and TSNAs were quantified by isotope-dilution liquid chromatography-tandem mass spectrometry (LC-MS/MS). The nicotine method is described elsewhere and had a limit of quantification (LOQ) of 0.20 ng/sample or a maximum of 0.01 ng/g dust (all units specified as ng/g refer to ng/g sieved dust) (G. E. Matt et al., 2021a,b). TSNAs are reported as the sum of 4-(methylnitrosamino)-1-(3-pyridyl)-1-butanone (NNK); *N*′-nitrosonornicotine (NNN); *N*-nitrosoanatabine (NAT), and *N*-nitrosoanabasine (NAB). The TSNA method is described elsewhere and had an individual TSNA LOQ of 1.25 ng/sample or a maximum of 0.26 ng/g (Matt et al., 2016). Pb, Cd, and As in dust were quantified by inductively coupled plasma-mass spectrometry (ICP-MS) as described elsewhere (Matt et al., 2021, 2021b). The LOQ for Pb was 50 ng/sample or a maximum of 2.74 ng/g, while Cd and As had a LOQ of 25 ng/sample or a maximum of 1.37 ng/g (details on supplementary information). NIST Standard Reference Material 1640a (Trace elements in Natural Water) was used to verify the accuracy of the quantification of Pb (95.54 % accuracy), Cd (99.47 % accuracy), and As (101.19 % accuracy). All measurements of nicotine, Pb, Cd, As, and TSNA were above their respective LOQ.

### 2.3. Data analysis

To address positively skewed distributions, we applied logarithmic transformations to all the Pb, Cd, As, nicotine, and TSNAs dust loadings. Pearson correlations with Bonferroni-correction were used to assess the bivariate associations between Pb, Cd, As, nicotine, and TSNAs. The associations between contaminant markers in dust and socio-demographic, housing, and smoking characteristics were examined using linear regression models. We used log-transformed loadings of the environmental markers (Pb, Cd, As, TSNAs, and nicotine) to test the association with sociodemographic characteristics, followed by their associations with home structural characteristics and smoking patterns. Finally, variables demonstrating a significant association with contaminant markers were selected to build multivariable regression models (final sample size n = 179) that evaluated the association of Pb, Cd, As, and TSNAs with nicotine, the main THS marker. Finally, based on these models, we estimated the mean dust loading of Pb, Cd, and As for homes free of tobacco smoke pollution (i.e., nicotine per m^2^ < LOD). We then calculated the percent reduction in mean dust Pb, Cd, and As loadings that could theoretically be achieved if homes had been 100 % tobacco-free. All statistical analyses were conducted using R (version 4.3.2) and SPSS (version 28).

## 3. Results

### 3.1. Distribution of metals and THS markers in settled house dust

**Fig. 1.**
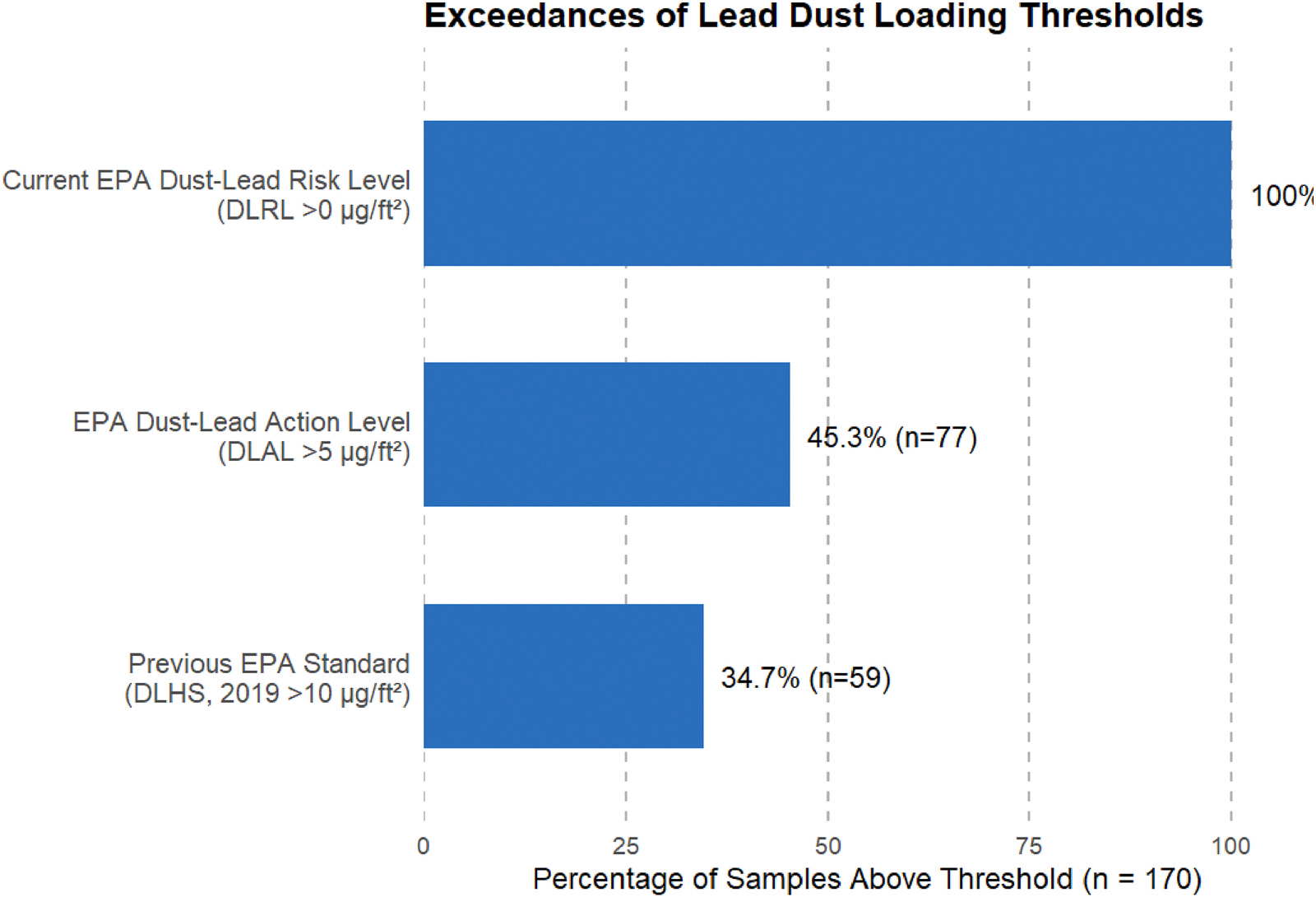
Homes with levels above the EPA standards for lead loading in dust. Abbreviations: DLRL: Dust-Lead Reportable Level; DLAL: Dust-Lead Action Level; DLHS: Dust-Lead Hazard Standards.

Pb, Cd, As, and nicotine were detected in all the collected floor dust samples, and 84.2 % of the samples had detectable levels of TSNAs (see Table 1 for additional information). As shown in Fig. 1, all dust samples (100 %) collected from the study homes exceeded the current EPA Dust-Lead Reportable Level (DLRL >0 μg/ft^2^), indicating that every sampled residence had detectable lead in floor dust. Furthermore, nearly half of the homes (45.3 %) had lead loadings above the EPA Dust-Lead Action Level (DLAL >5 μg/ft^2^), a threshold that may warrant public health intervention. Additionally, 34.7 % of the samples exceeded the previous EPA standard (DLHS >10 μg/ft^2^), which was in effect prior to the 2024 updates (EPA, 2024).

**Fig. 2.**
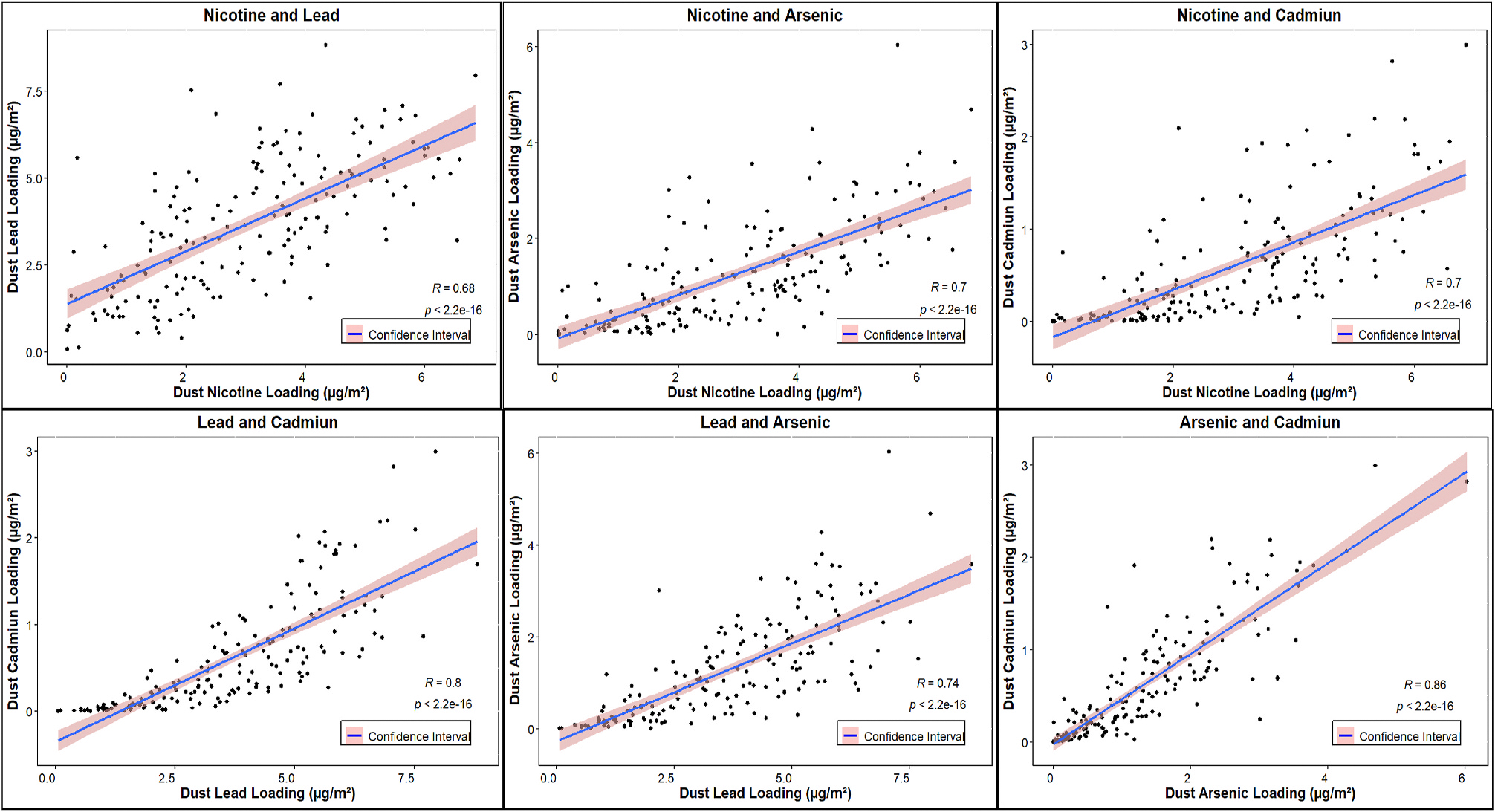
Linear associations between metal and nicotine dust loadings (log-transformed).

As presented in Fig. 2, nicotine loading was significantly (Bonferroni *p* < 0.001) correlated with Pb, Cd, and As loadings (r = 68; r = 0.70; r = 0.70, respectively). All metals in dust were correlated with each other, as Pb loading was significantly (Bonferroni *p* < 0.001) correlated with As and Cd (r = 0.74; r = 0.80 respectively), while Cd loading was significantly (Bonferroni *p* < 0.001) correlated with As loading (r = 0.86). In addition, as presented in Fig. 1S, the levels of TSNAs in dust were significantly correlated with the dust loadings of nicotine, Pb, Cd, and As (r = 78; r = 0.50; r = 0.51; r = 0.47, respectively).

### 3.2. Bivariate associations between metals and THS markers with sociodemographic/home characteristics and smoking behaviors

We found no significant associations between Pb, Cd, and nicotine with sociodemographic factors (Table 1S). However, households with income greater than $15,000 had significantly higher As dust loading than households with an income of ≤$15,000 (geomean: 2.48 μg/m^2^ vs 1.42 μg/m^2^, *p* = 0.025). Also, black children lived in homes with significantly lower As dust loading than homes of White children (geomean: 1.22 μg/m^2^ vs 1.42 μg/m^2^, *p* = 0.016) (Table 1S). Regarding building structure characteristics and smoking behaviors presented in Table 2S, we found that Pb dust loading was significantly associated with the age of the homes, as homes built after the year 1960 had significantly lower Pb loading than homes built before 1960 (geomean: 23.9 μg/m^2^ vs 98.1 μg/m^2^, *p* < 0.001). The year 1960 was selected as a pivotal year because, according to the EPA, homes built before this year are five times more likely to contain lead compared to newer homes (EPA, 2023). Additionally, homes with larger square footage (>1500 ft^2^) had significantly higher levels of Pb loading in dust compared to homes <800 ft^2^ (geomean: 80.3 μg/m^2^ vs 28.0 μg/m^2^, *p* = 0.045). Similarly, homes with a square footage >1500 ft^2^ had significantly higher levels of As loading compared to homes <800 ft^2^ (geomean: 2.51 μg/m^2^ vs 0.67 μg/m^2^, *p* = 0.007). Cd loading in dust was not associated with any home characteristics or smoking behaviors. Nicotine loading was significantly associated with a smoking ban, as homes with an indoor smoking ban had lower nicotine dust levels than homes with no indoor smoking ban (geomean: 7.4 μg/m^2^ vs 21.7 μg/m^2^, *p* = 0.004).

### 3.3. Factors affecting metals loading in dust

The multivariable regression models examining associations between the metal and nicotine loadings, controlling for home and smoking characteristics, showed overall statistically significant associations between nicotine and Pb (R^2^ = 0.6191; F(10, 168) = 27.31, *p* < 0.001), Cd (R^2^ = 0.6757; F(10, 168) = 35.00, *p* < 0.001), and As (R^2^ = 0.6185; F(10, 168) = 27.24, *p* < 0.001). As presented in Table 2, increasing nicotine loading was associated with higher levels of Pb loading ($\hat{\beta }\;=\;0.681$, *p* < 0.001), Cd loading ($\hat{\beta }\;=\;0.706$, *p* < 0.001), and As loading ($\hat{\beta }\;=\;0.689$, *p* < 0.001). Homes built after 1960 ($\hat{\beta }\;=\;-1.02$, *p* < *0.001*) had lower Pb dust loading than older homes. The semi-partial r^2^ showed that nicotine loading uniquely accounted for 43 %, the year a house was built 4 %, and home size 3 % of the variance in Pb dust loading. Similarly, nicotine loading uniquely accounted for 58 % of the variance in Cd and 52 % in As dust loading, while housing type and home size contributed to 2 % and 1.5 % of the variance for As dust loading (Table 2).

**Fig. 3.**
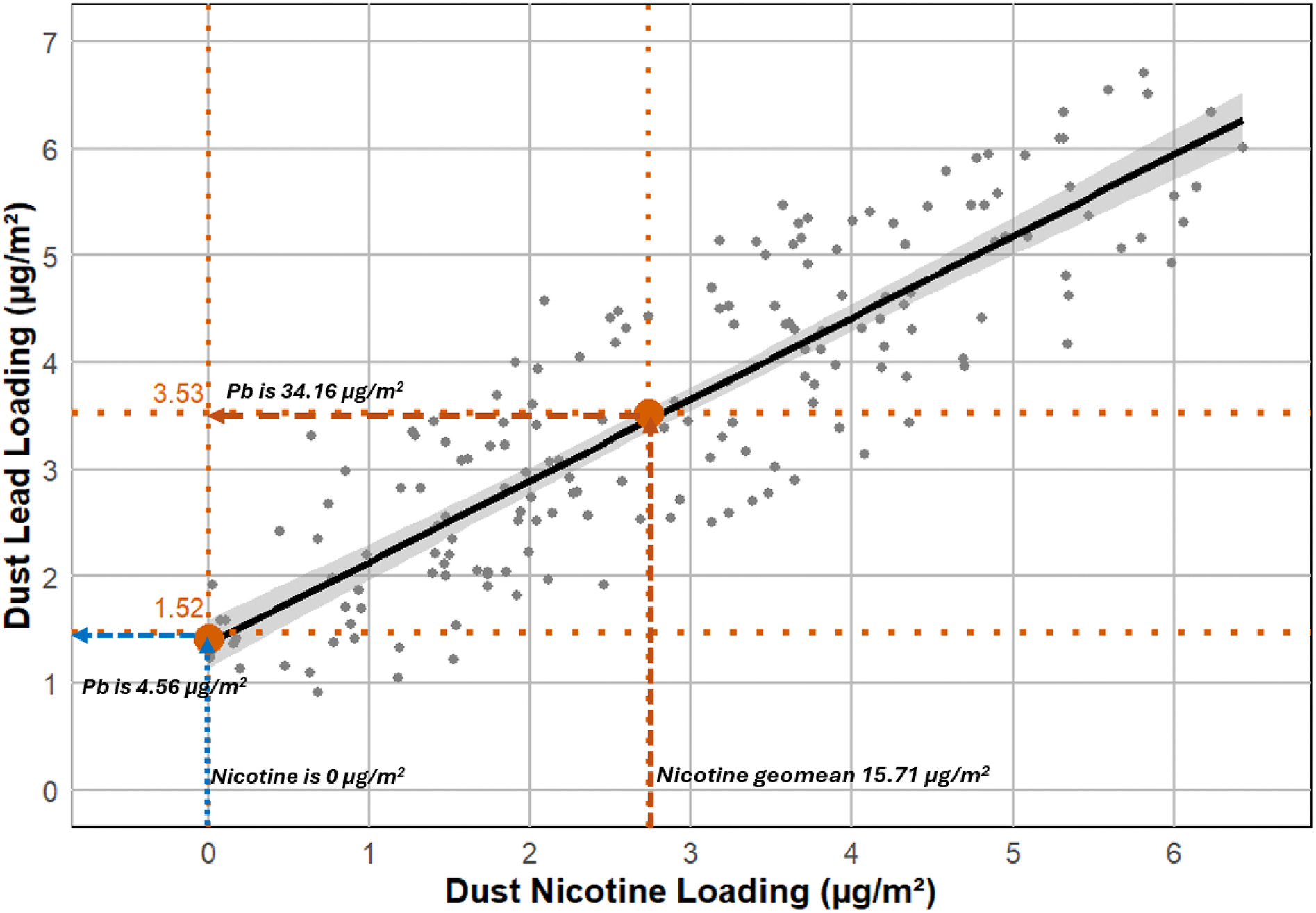
Linear association between nicotine and lead (log-transformed) and projected lead dust loading for nicotine-free homes. Note: gray shaded area corresponds to 95 % Confidence Interval.

To estimate how much Pb, Cd, and As levels would be reduced in homes without thirdhand smoke pollution, we used the multivariable models to project mean Pb, Cd, and As dust loadings for homes with 0 μg/m^2^ of dust nicotine. After controlling for building characteristics and smoking behaviors, the projected Pb, Cd, and As loadings were 4.56 μg/m^2^ (CI: 3.12, 6.69), 0.20 (CI: 0.15, 0.99), 0.98 (CI: 0.78, 1.23), respectively. The estimated reduction of Pb loading when nicotine is zero is illustrated in Fig. 3. In a hypothetical nicotine-free home, this represents 87 %, 49 %, and 38 % reductions of the mean Pb, Cd, and As loading, respectively. The results of the TSNA associations are presented in the supplementary material. Briefly, after controlling for the home and smoking characteristics, TSNAs dust loading was significantly associated with Pb, Cd, As, and nicotine loading in the dust (Tables 3S). As presented in Table 3S, for a unit increase of TSNAs loading in dust, the studied metals increased significantly: Pb loading ($\hat{\beta }\;=\;0.623$, *p* < 0.001), Cd loading ($\hat{\beta }\;=\;0.274$, *p* < 0.001), and As ($\hat{\beta }\;=\;0.431$, *p* < 0.001). Also, as shown in Table 4S, increasing nicotine loading was associated with higher levels of TSNAs loading ($\hat{\beta }\;=\;0.826$, *p* < 0.001).

## 4. Discussion

### 4.1. Associations between THS markers and heavy metals in settled dust

Settled house dust is a known reservoir for THS, the toxic mixture of tobacco smoke pollutants that can persist in indoor environments for years, and dust nicotine is a specific marker for THS pollutants. This is the first study to show that after controlling for building (e.g., age) and resident characteristics (e.g., smoking behavior, smoking ban), a large proportion of the variance in Pb, Cd, and As levels was attributable to tobacco use. Approximately 60 % of the variance in the heavy metal dust loading was accounted for by nicotine dust loading. In our models, hypothetical homes without any tobacco smoke residue were projected to have 82 % lower mean Pb, 49 % lower mean Cd, and 38 % lower mean As loadings compared to those observed in the studied homes, even after accounting for building characteristics and smoking behaviors. Also, the strong associations between TSNAs and metal levels further support tobacco smoke as a major, yet underrecognized, source of toxic metals in indoor environments.

All sampled homes (100 %) in this study exceeded the EPA's newly proposed Dust-Lead Reportable Level (DLRL >0 μg/ft^2^), indicating that every residence tested contained detectable levels of lead in floor dust. Furthermore, 45.3 % of homes exceeded the current EPA Dust-Lead Action Level (DLAL >5 μg/ft^2^), while 34.7 % surpassed the previous EPA hazard and clearance standard (DLHS >10 μg/ft^2^). Under the most recent federal guidance, even the presence of any detectable lead dust is considered hazardous in certain residential settings, meaning that all homes in this sample would fail to meet current indoor safety standards (EPA, 2024). Beyond these regulatory thresholds, which are established to protect human health, the toxicological evidence further underscores the seriousness of this issue.

High levels of Pb in dust can pose a risk, especially to children who are more susceptible to its adverse effects due to their developing bodies and behaviors, such as hand-to-mouth contact (Roberts et al., 2009). From a toxicological perspective, the higher Pb concentrations observed in THS samples have serious implications for cumulative exposure and potential adverse health outcomes. Yet even at low doses, chronic Pb exposure has been shown to impair cognitive function, disrupt neurodevelopment, and affect renal physiology (Lanphear et al., 2000; Hong et al., 2014; Satarug et al., 2020). Given that children typically ingest more dust, and inhale and absorb more Pb than adults, elevated Pb levels in THS-contaminated dust represent a significant and realistic exposure pathway that warrants urgent attention (Özkaynak et al., 2011; Sample, 2024). These toxicological insights demonstrate the significance of the elevated Pb levels observed in THS samples and the need for targeted remediations, particularly in homes with children.

The dust loading levels of Pb and Cd in our study are similar to the levels found in Matt et al., 2021a,b (Geomean: Pb: 31.4 μg/m^2^, Cd: 1.6 μg/m^2^) (Matt et al., 2021, 2021b). The Pb concentrations in household floor dust in the present study (mean: 186.9 μg/g, Table 5S), are higher than those reported in a recent meta-analysis of non-superfund U.S. homes (mean: 153 μg/g) surveyed from 1996 to 2016 (Frank et al., 2019). The results of our study suggest that decades of permissive indoor smoking policies have had a strong and lasting impact on the pollution of indoor environments with heavy metals. These findings underscore the widespread nature of lead contamination in household dust from THS and emphasize the need for urgent efforts to identify and mitigate contributing sources, particularly in communities already facing environmental health disparities (Hamzai et al., 2024).

The significant correlations found in this study between nicotine and the Pb and Cd loadings in settled dust are consistent with the findings from Matt et al. (2021a,b). Willers et al. (2005) reported a more modest association between nicotine and lead concentration in fine dust and no significant associations between nicotine and metals in coarse dust (Willers et al., 2005). This may be due to the different dust sampling methods used, as Willers et al. (2005). collected dust from the bags of participants’ vacuum cleaners, while a cyclone-equipped vacuum cleaner was utilized for our study, allowing for the more efficient collection of fine particles and a dust sample taken from a known area (Farfel et al., 1994). We also sieved our samples to dust particles smaller than 150 μm. Additionally, our study had a lower detection limit for metals and stronger statistical power. To our knowledge, this is the first study to find a correlation between As and nicotine loading in settled dust of smoker homes. The As loading levels in dust found in this study were higher than in homes located near mining sites in the U.S. (this study median: 3.14 μg/m^2^ vs 1.32 μg/m^2^) listed as Superfund sites by the US EPA National Priorities List (Beamer et al., 2016).

The significant contribution of the age of the home to lead dust loading found in this study, though smaller than dust nicotine levels, is likely the result of lead-based paints use in older homes. Although lead in paint was banned in 1978, the prevalence of lead in homes increases significantly with the age of the building. According to the US EPA, 69 % of homes built in the U.S. before 1960 contain lead-based paint (EPA, 2023). Among the 112/170 homes in this study with available construction year data and detectable lead dust loading above the current EPA DLRL (0 μg/ft^2^), 64 (57 %) were built prior to 1960. This indicates a potential contribution from lead paint as well as tobacco smoking to Pb hazards in these homes. Other home characteristics, such as a residence with larger square footage or a single-unit house, exhibited a significant positive association with dust loading of As and Pb. This association may be attributed to factors potentially present in larger single-unit homes, such as increased access to outdoor areas like backyards, which could introduce soil-related contaminants into the indoor environment (Palma-Lara et al., 2020). Also, as home square footage increases, the likelihood of accommodating more smokers also rises, fostering a common phenomenon of cohabitation among individuals who engage in smoking behavior.

### 4.2. Insights and limitations

Our findings indicate that dust nicotine loading has a strong association with the levels of metals in household dust. The correlation analyses and regression models revealed significant associations between nicotine, a marker of THS, and the levels of Pb, Cd, and As in dust, suggesting that smoking is a primary contributor to the accumulation of these metals in indoor environments. In addition, when these associations were adjusted for other factors that may contribute to the loading of metals in floor dust (e.g., building year as a proxy for the presence of lead-based paint), the association between nicotine and metals remained dominant. This indicates that smoking is a significant source of these metals in household floor dust, controlling for other confounding factors evaluated in this study. Our study findings underscore the significant impact of complete building-wide indoor smoking bans and the removal of THS pollutant reservoirs on reducing metal concentrations in homes. The projected reductions hold true irrespective of other confounding factors, emphasizing the pivotal role of THS as an indoor pollutant.

A limitation of this study is that we did not evaluate external factors such as proximity to industrial activities, construction materials, or vehicle emissions that could be potential sources of metals in indoor dust. This gap may help explain the unexpected positive association between reported smoking bans and cadmium and arsenic loading. Although tobacco smoke is a known source of these metals, cadmium and arsenic also arise from non-smoking sources, including outdoor industrial emissions, soil and dust infiltration, and legacy contamination, which can elevate indoor levels regardless of smoking behavior. Also, we did not assess other behavioral factors in the use of products that may release some of these metals. For example, As is known to be present in various human-induced sources such as pesticides and other commercial/industrial products that were not assessed in this study (Ali et al., 2021). However, these external factors offer alternative third-variable explanations only if they are also associated with tobacco use, such as if homes with high levels of dust nicotine were more likely to be in areas of industrial activity that contributed to heavy metal pollution. To further investigate the plausibility of this alternative explanation, we examined six homes that were located in a ZIP code known for industrial pollution. We found that their Pb levels were similar or lower than homes located outside of this ZIP code.

Environmental tobacco smoke is likely a large and unrecognized contributor to toxic metals in dust. In October 2024, the U.S EPA strengthened requirements for the removal of Pb-based paint hazards in buildings (EPA, 2024). The plan focuses on reducing indoor Pb exposure through abatement activities to advance environmental justice, as deteriorated Pb-based paint is more likely to be found in lower-income communities. Strikingly, our findings indicate that over four decades after the phase-out of lead in paint and gasoline, tobacco use may exert a stronger influence on indoor lead dust loading than these historically dominant sources. This underscores the need to re-evaluate contemporary exposure pathways that have been largely overlooked in lead risk assessments. Also, disparities related to THS pollution suggest that Pb, Cd, and As related to THS are also an environmental justice issue, as THS levels are higher in low-income homes and in disadvantaged minorities (Matt et al., 2022). By demonstrating that THS pollution is associated with elevated levels of multiple toxic metals in dust, especially in low-income and minority households, this study positions THS not just as a tobacco exposure problem but as a broader environmental justice and toxic metal contamination issue. Our findings challenge the assumption that Pb in dust is primarily from legacy sources (e.g., paint, gasoline), showing that current or past tobacco use may contribute more significantly to Pb dust loading than the age of the home in many cases. This reframes tobacco-related metal exposure as an urgent and ongoing public health issue, especially in older homes. Thus, future strategies to reduce exposure to Pb and other toxic metals should incorporate comprehensive tobacco control measures as a core component of environmental health interventions. These efforts should include health education campaigns that raise awareness about the toxic legacy of tobacco use, including its role in contributing to indoor contamination through both active smoking and thirdhand smoke (THS) residue (Matt et al., 2023). Taken together, these findings suggest that environmental tobacco smoke may be one of the most pervasive and underappreciated sources of toxic metal contamination in residential dust today.

## Supplementary Material

1

## Figures and Tables

**Table 1:** Descriptive statistics of contaminant dust loading.

Contaminant Marker	n	Mean (SD)	GM	95 % CI	Min	Q1	Median	Q3	Max	% > LOD

Dust Lead Loading (μg/m^2^)	179	202.66 (605.5)	34.16	113.4–291.9	0.070	6.71	35.24	166.7	6734.0	100
Dust Arsenic Loading (μg/m^2^)	179	8.13 (32.76)	1.58	3.30–12.9	0.003	0.47	1.85	5.86	414.2	100
Dust Cadmium Loading (μg/m^2^)	179	1.36 (2.4)	0.39	1.01 –1.75	0.001	0.11	0.49	1.45	18.9	100
Dust Nicotine Loading (μg/m^2^)	179	73.77 (140.7)	15.71	53.0–94.5	0.010	4.50	21.76	66.3	940.6	100
Dust Total TSNAs Loading (ng/m^2^)^a^	158	51.95 (155.5)	5.96	27.5–76.4	0.008	1.40	9.94	46.5	1743.2	84.2

a Results include imputed TSNAs values of 0.025 ng/g for the < LOD values.

Abbreviations: SD, standard deviation; GM, geometric mean; LOD, limit of detection.

**Table 2:** Multivariable regression models assessing the association between metal dust loading and dust nicotine loading, controlling for home and smoking characteristics (*n* = 179).

*Model Fit*	Lead Loading (μg/m^2^)				Cadmium Loading (μg/m^2^)				Arsenic Loading (μg/m^2^)			
	R2 = 0.619 (p < 0.001)				R^2^ = 0.676 (p < 0.001)				R^2^ = 0.618 (p < 0.001)			
	$\hat{\beta }$	Semi-Partial R^2^	95 % CI	P-value	$\hat{\beta }$	Semi-Partial R^2^	95 % CI	P-value	$\hat{\beta }$	Semi-Partial R^2^	95 % CI	P-value

**Nicotine Loading (μg/m^2^)**	0.681	0.431	[0.58; 0.78]	**<0.001**	0.706	0.583	[0.62; 0.78]	**<0.001**	0.689	0.518	[0.60; 0.78]	**<0.001**
**Housing type**												
Single-family	Ref				Ref				Ref			
Multi-unit apartment	−0.387	0.006	[−0.86; 0.09]	0.110	−0.246	0.003	[−0.64; 0.14]	0.215	−0.599	0.017	[−1.04; −0.16]	**0.008**
**Year House Built** ^a^												
≤ 1960	Ref				Ref				Ref			
> 1960-present	−1.002	0.040	[−1.52; −0.48]	**<0.001**	−0.005	0.001	[−0.43; 0.43]	0.981	0.082	0.001	[−0.40; 0.56]	0.132
Missing (n = 67)	−0.889		[−1.44; −0.34]	**0.002**	−0.144		[−0.59; 0.31]	0.533	0.125		[−0.38; 0.63]	0.626
**House Size** ^b^												
<800 ft^2^	Ref				Ref				Ref			
800–1500 ft^2^	−0.177	0.027	[−0.77; 0.41]	0.554	0.184	0.017	[−0.30; 0.67]	0.454	0.511	0.015	[−0.03; 1.05]	0.066
>1500 ft^2^	0.218		[−0.48; 0.91]	0.537	0.336		[−0.24; 0.91]	0.251	0.748		[0.10; 1.40]	0.023
Missing (n = 38)	−0.923		[−1.58; −0.26]	**0.007**	−0.456		[−1.00; 0.09]	0.102	0.126		[−0.03; 0.74]	0.686
**Income** ^c^												
≤ $15,000	Ref				Ref				Ref			
> $15,000	0.152	0.001	[−0.28; 0.59]	0.496	0.217	0.003	[0.14; 0.58]	0.239	0.378	0.008	[−0.03; 0.78]	0.067
**Smoking Ban at Home**												
No	Ref				Ref				Ref			
Yes	0.383	0.009	[−0.09; 0.87]	0.119	0.662	0.026	[0.27; 1.05]	**0.001**	0.561	0.016	[0.11; 1.06]	**0.014**
Missing (n = 39)	0.478		[−0.07; 1.02]	0.088	0.576		[0.12; 1.03]	**0.013**	0.454		[−0.05; 0.96]	0.079

Abbreviations: β, estimate change; CI, confidence interval; Ref, reference group. P-value refers to adjusted multiple linear regression model results with nicotine (log-transformed) as the main predictor variable adjusted for other covariates and the log-transformed contaminant marker concentration as the response variable. Bold font indicates statistical significance at p < 0.05 for each parameter.

a Based on previous national surveys across the U.S., which indicate that most (69 %) homes built before 1960 can have a presence of Pb due to the use of lead-based paint.

b The home size corresponds to the square footage of the participants’ homes.

c Home Smoking Ban: Smoking is never allowed inside the home.

## Data Availability

Data will be made available on request.

## HIGHLIGHTS

- Household tobacco use as a main source of toxic metals in home dust.
- Lead was detected in all homes, exceeding the new EPA dust-lead reportable level.
- Metals in home dust correlated with nicotine and tobacco-specific nitrosamines.
- Projection: tobacco-free homes could lower Pb, Cd, and As by 87 %, 49 %, & 38 %.

## Data sharing

Data included in this study are not publicly available. However, requests for access to de-identified information should be sent to Dr. E. Melinda Mahabee-Gittens (Melinda.Mahabee-gittens@cchmc.org).

## Appendix A. Supplementary data

Supplementary data to this article can be found online at https://doi.org/10.1016/j.chemosphere.2025.144820.
