# Supplementary material for: Environmental tobacco smoke is a major contributor to lead, cadmium, and arsenic in settled house dust
Source: Chemosphere. Author manuscript; Available in PMC 2026 Jul 12. (PMC13356893; doi:10.1016/j.chemosphere.2025.144820)
Supplement: 1 [file NIHMS2189185-supplement-1.docx]

**Supplementary Materials**

In Figure 1S, we present the correlations between TSNAs and other analytes (nicotine, cadmium, arsenic, and lead). Table 1S describes the associations between socio-demographic characteristics and the contaminant dust loading of Pb, Cd, and nicotine. Table 2S presents the building structure characteristics and smoking behaviors, highlighting that homes built before 1960 have significantly lower Pb loading levels. Table 3S shows the increase in TSNAs loading in dust in relation to the significant increase of the studied metals. Additionally, Table 4S presents the association between nicotine and TSNAs loading.


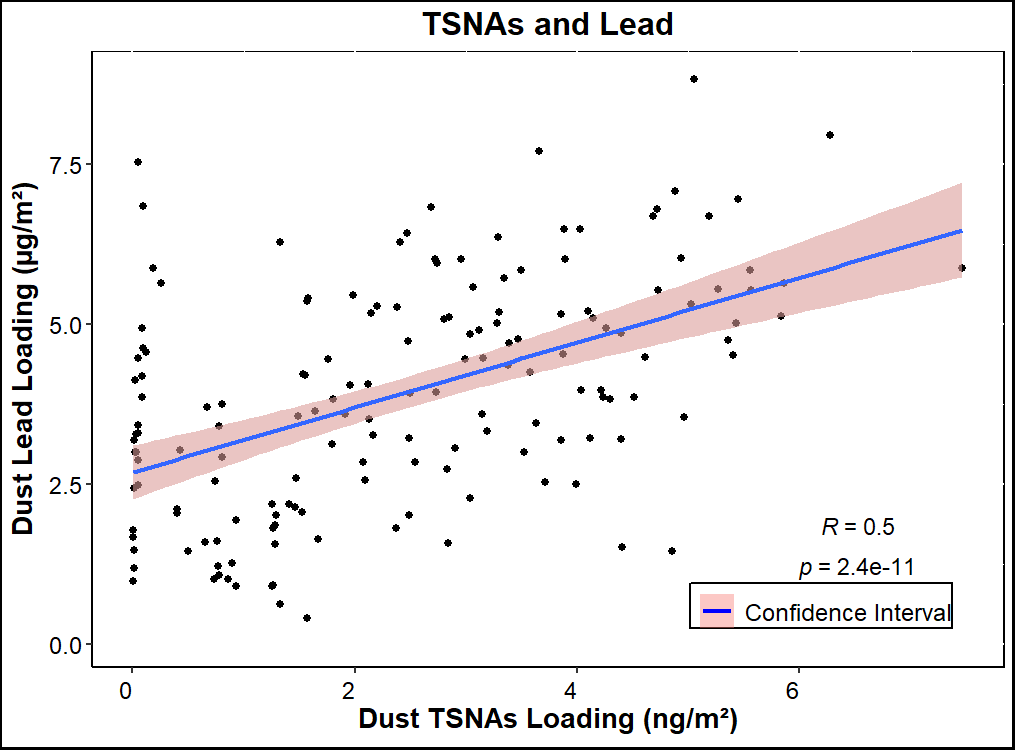

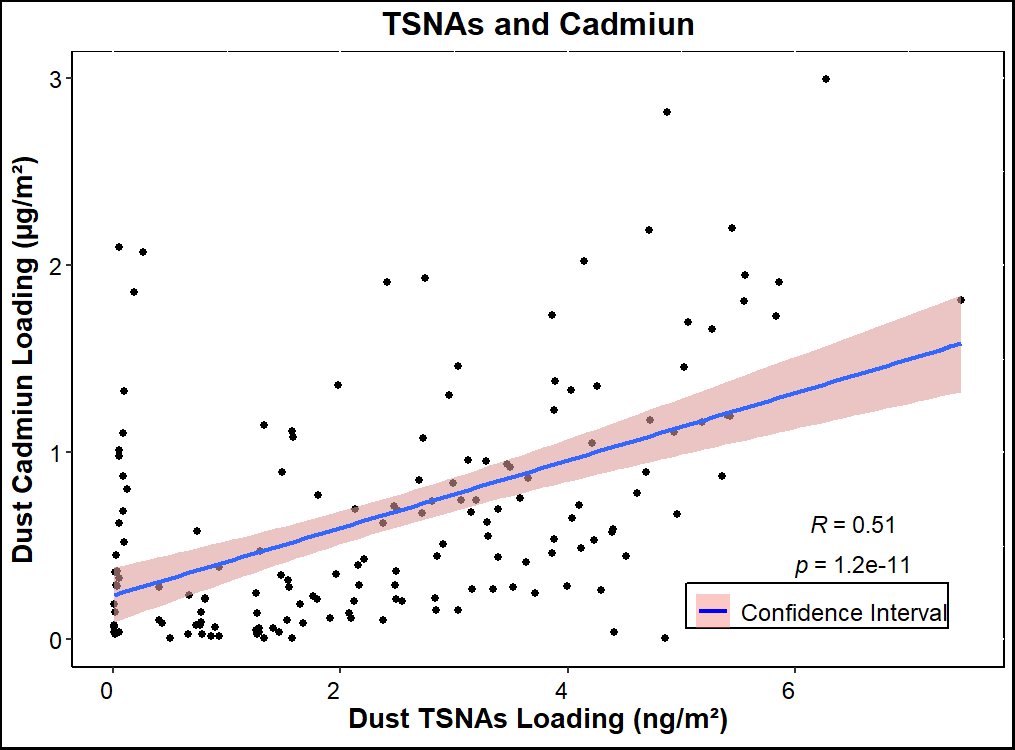

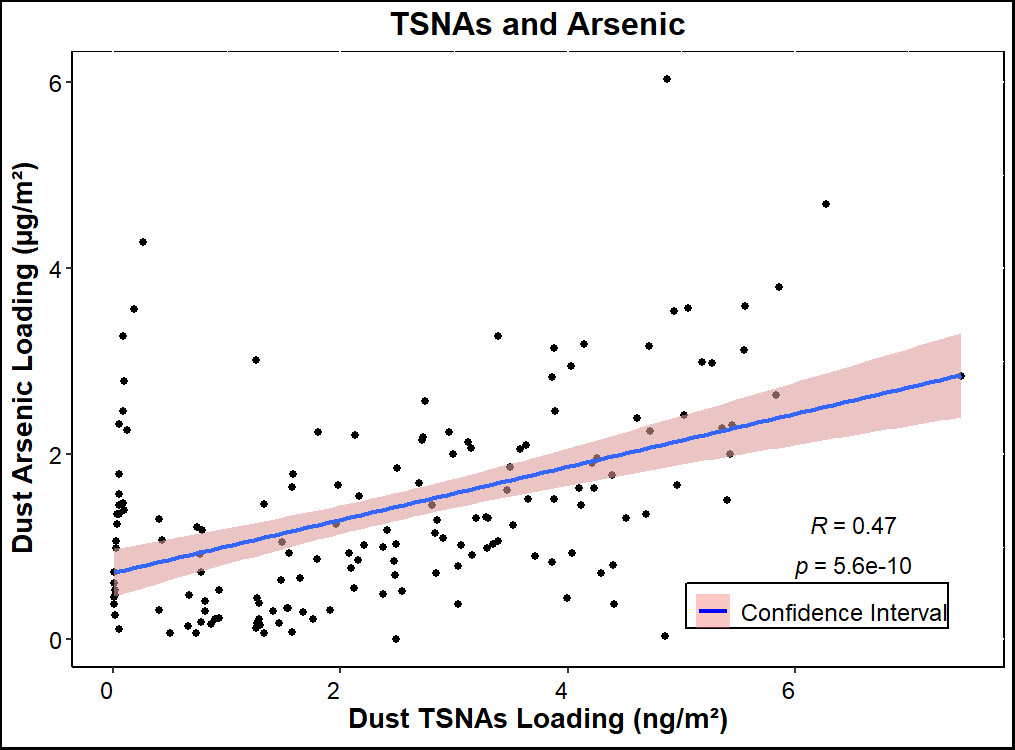

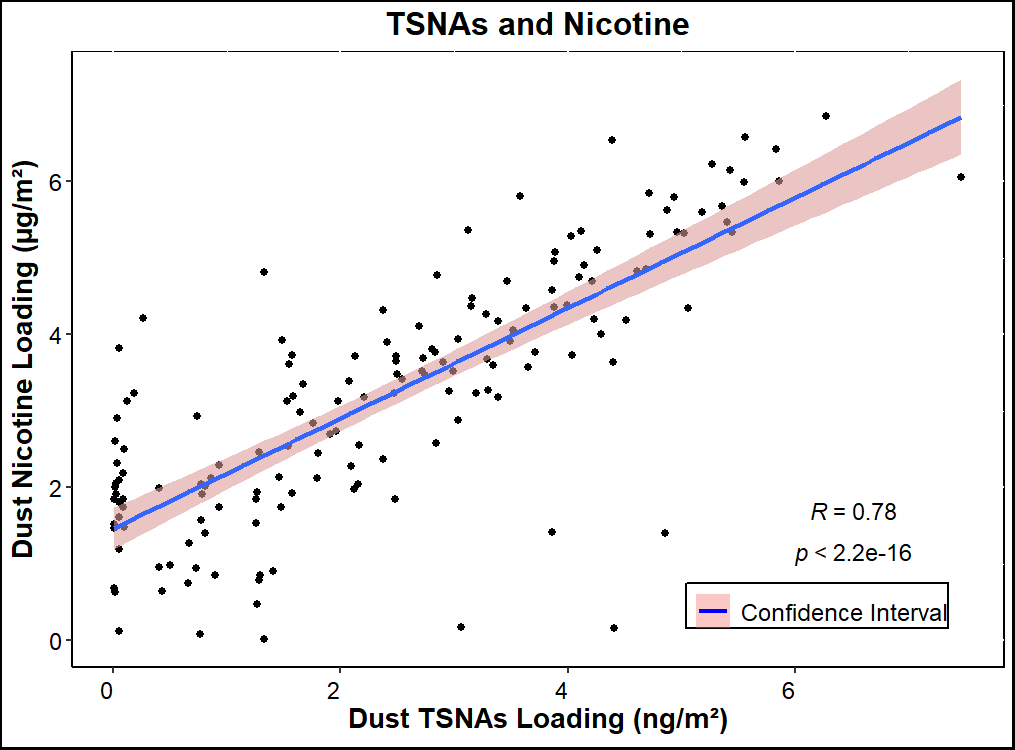


**Figure 1S. Linear associations between metal and TSNAs dust loadings (log-transformed).**

| **Table 1S.**  **Summary of** **Socio-Demographic Characteristics and Contaminant Dust Loading Differences (N=179)** | | | | | | | | | | | | | | | | |  | |  |
| --- | --- | --- | --- | --- | --- | --- | --- | --- | --- | --- | --- | --- | --- | --- | --- | --- | --- | --- | --- |
|  | **Overall** **^a^**  ***n* (%)** | | **Lead (µg/m^2^)** | | | | **Cadmium (µg/m^2^)** | | | | **Arsenic (µg/m^2^)** | | | | **Nicotine (µg/m^2^)** | |  | |  |
|  |  |  | **GM (CI)** | | **p-value** | | **GM (CI)** | | **p-value** | | **GM (CI)** | | **p-value** | | **GM (CI)** | | **p-value** | |  |
| **Household Income** |  | |  | |  | |  | |  | |  | |  | |  | |  | |  |
| ≤ $15,000 | 118 (66) | | 30.6 [20.6; 45.4] | | Ref | | 0.45 [0.001; 18.9] | | Ref | | 1.26 [0.87; 1.80] | | Ref | | 15.9 [10.5; 23.9] | | Ref | |  |
| > $15,000 | 61 (44) | | 42.4 [26.0; 69.0] | | 0.320 | | 0.54 [0.02; 15.76] | | 0.155 | | 2.48 [1.58; 3.87] | | **0.025** | | 15.4 [9.68; 24.4] | | 0.956 | |  |
| **Parent Education** |  | |  | |  | |  | |  | |  | |  | |  | |  | |  |
| ≤ High school | 93 (52) | | 35.9 [22.6; 56.8] | | Ref | | 0.40 [0.27; 0.59] | | Ref | | 1.66 [1.09; 2.53] | | Ref | | 18.3 [12.0; 27.9] | | Ref | |  |
| >College | 86 (14) | | 32.4 [21.5; 48.9] | | 0.748 | | 0.38 [0.26; 0.56_ | | 0.858 | | 1.50 [1.02; 2.21] | | 0.725 | | 13.3 [8.41; 21.2] | | 0.356 | |  |
| **Child Sex** |  | |  | |  | |  | |  | |  | |  | |  | |  | |  |
| Male | 96 (54) | | 32.3 [21.3; 49.1] | | Ref | | 0.42 [0.28; 0.62] | | Ref | | 1.85 [1.23; 2.79] | | Ref | | 18.4 [12.3; 27.6] | |  | |  |
| Female | 83 (46) | | 36.4 [23.0; 57.8] | | 0.701 | | 0.35 [0.24; 0.52] | | 0.524 | | 1.32 [0.89; 1.95] | | 0.239 | | 13.1 [8.05; 21.2] | | 0.276 | |  |
| **Child Race** |  | |  | |  | |  | |  | |  | |  | |  | |  | |  |
| White | 56 (31) | | 43.9 [27.9; 59.1] | | Ref | | 0.58 [0.39;0.87] | | Ref | | 2.61 [1.73; 3.94] | | Ref | | 17.8 [10.8; 29.4] | | Ref | |  |
| Black | 105 (59) | | 32.1 [20.7; 49.7] | | 0.363 | | 0.33 [0.23; 0.48] | | 0.066 | | 1.22 [0.83; 1.80] | | **0.016** | | 15.0 [ 9.97; 22.6] | | 0.625 | |  |
| Other | 18 (10) | | 22.5 [7.84; 64.6] | | 0.237 | | 0.29 [0.09; 0.84] | | 0.159 | | 1.54 [0.51; 4.62] | | 0.304 | | 13.8 [3.51; 54.5] | | 0.660 | |  |
|  |  | |  | |  | |  | |  | |  | |  | |  | |  | |  |
| **Child Age,**  **M (SD)** | 6.0 (4.9) | | *r* = 0.027 | | 0.378 | | *r* = 0.023 | | 0.413 | | *r* = 0.011 | | 0.713 | | *r* =0.037 | | 0.246 | |  |
| 0-1 years | 37 (20) | | 28.8 [14.4; 57.7] | | Ref | | 0.35 [0.19; 0.65] | | Ref | | 1.55 [0.83; 2.90] | | Ref | | 11.5 [5.43; 24.3] | | Ref | |  |
| 2-4 years | 30 (17) | | 28.3 [14.5; 55.4] | | 0.976 | | 0.35 [0.18; 0.68] | | 0.990 | | 1.50 [0.77; 2.93] | | 0.939 | | 13.1 [5.54; 31.0] | | 0.801 | |  |
| 5-9 years | 62 (35) | | 41.6 [24.6; 70.4] | | 0.398 | | 0.41 [0.26; 0.64] | | 0.716 | | 1.70 [1.07; 2.71] | | 0.822 | | 19.3 [12.2; 30.5] | | 0.238 | |  |
| 10-17 years | 50 (28) | | 34.0 [17.8; 65.0] | | 0.715 | | 0.42 [0.24; 0.76] | | 0.662 | | 1.52 [0.82; 2.81] | | 0.958 | | 17.1 [9.0; 32.3] | | 0.388 | |  |
| Abbreviations: GM, geometric mean; CI, confidence interval; Ref, reference group; M, mean; SD, standard deviation; r, Pearson correlation coefficient. ^a^ n (%) unless otherwise noted. P-values are unadjusted and refer to simple linear regression model results with one sociodemographic characteristic as the explanatory variable and a marker in log scale as the response variable. Bold font indicates statistical significance at p < 0.05 based on linear regression. | | | | | | | | | | | | | | | | | | |  |
| **Table 2S. Summary of Home Structure Features, Smoking Habits, and Differences in Contaminant Dust Levels** | | | | | | | | | | | | | | | | | |  | |
|  | | **Overall  ^a^**  ***n* (%)** | | **Lead (µg/m^2^)** | | | | **Cadmium (µg/m^2^)** | | | | **Arsenic (µg/m^2^)** | | | | **Nicotine (µg/m^2^)** | | | |
|  | |  |  | **GM (CI)** | | **p-value** | | **GM (CI)** | | **p-value** | | **GM (CI)** | | **p-value** | | **GM (CI)** | | **p-value** | |
| **Home type** | |  | |  | |  | |  | |  | |  | |  | |  | |  | |
| Single-family | | 66 (37) | | 47.4 [29.0; 77.3] | | Ref | | 0.47 [0.29; 0.77] | | Ref | | 2.35 [1.44; 3.83] | | Ref | | 12.0 [6.74; 21.0] | | Ref | |
| Multi-unit apartment | | 113 (63) | | 28.2 [19.0; 41.9] | | 0.108 | | 0.35 [0.25; 0.48] | | 0.2952 | | 1.26 [0.89; 1.77] | | **0.035** | | 18.5 [12.8; 26.7] | | 0.198 | |
| **Year Home Built (n=112)** ^b^ | |  | |  | |  | |  | |  | |  | |  | |  | |  | |
| ≤ 1960 | | 64 (56) | | 98.1 [62.5; 154.0] | | Ref | | 0.59 [0.38; 0.92] | | Ref | | 2.20 [1.42; 3.40] | | Ref | | 21.4 [13.4; 34.2] | | Ref | |
| >1960-present | | 48 (43) | | 23.8 [14.0; 40.5] | | **0.001** | | 0.43 [0.26; 0.72]] | | 0.730 | | 1.77 [0.98; 3.20] | | 0.786 | | 13.3 [7.38; 24.0] | | 0.864 | |
| **House Size (n=141), M (SD)** ^c^ | | 1,430 (1,950) | | *r* = 0.534 | | 0.101 | | *r* = 0.229 | | 0.424 | | *r* = 0.357 | | 0.247 | | *r* =0.019 | | 0.956 | |
| <800 ft^2^ | | 31 (22) | | 28.0 [11.10; 70.8] | | Ref | | 0.25 [0.12; 0.54] | | Ref | | 0.67 [0.28; 1.60] | | Ref | | 10.3 [4.1; 25.9] | | Ref | |
| 800-1500 ft^2^ | | 77 (55) | | 42.1 [27.40; 64.7] | | 0.359 | | 0.51 [0.35; 0.74] | | 0.074 | | 2.17 [1.45; 3.24] | | **0.005** | | 16.9 [10.3; 27.9] | | 0.248 | |
| >1500 ft^2^ | | 33 (23) | | 80.3 [39.2; 164.0] | | **0.045** | | 0.60 [0.31; 1.15] | | 0.060 | | 2.51 [1.33; 4.76] | | **0.007** | | 17.9 [9.5; 34.0] | | 0.278 | |
| **Smoked every day in last 7 days** ^d^ | |  | |  | |  | |  | |  | |  | |  | |  | |  | |
| Yes | | 129 (72) | | 37.7 [26.4; 53.8] | | Ref | | 0.44 [0.39; 0.60] | | Ref | | 1.75 [1.24; 2.46] | | Ref | | 18.5 [12.8; 26.7] | |  | |
| No | | 50 (28) | | 26.5 [14.2; 49.3] | | 0.310 | | 0.29 [0.17; 0.50] | | 0.174 | | 1.23 [0.74; 2.03] | | 0.271 | | 10.3 [5.7; 18.5] | | 0.093 | |
| **No. of Cigarettes/day, M(SD)** ^e^ | | 8 (6.62) | | *r* = 0.005 | | 0.829 | | *r* = 0.011 | | 0.619 | | *r* = 0.001 | | 0.833 | | *r* = 0.024 | | 0.324 | |
| 1 - 5 Cigarettes | | 68 (39) | | 34.2 [19.4; 60.2] | | Ref | | 0.37 [0.24; 0.59] | | Ref | | 1.20 [0.73; 1.93] | | Ref | | 11.2 [6.49; 19.4] | | Ref | |
| 6 - 14 Cigarettes | | 69 (40) | | 30.5 [19.1; 48.6] | | 0.595 | | 0.33 [0.21; 0.50] | | 0.585 | | 1.52 [0.98; 2.36] | | 0.536 | | 18.9 [11.80; 30.0] | | 0.380 | |
| 15 - 40 Cigarettes | | 36 (21) | | 38.7 [20.9; 71.6] | | 0.546 | | 0.53 [0.29; 0.96] | | 0.787 | | 2.40 [1.31; 4.35] | | 0.975 | | 20.6 [10.50; 40.5] | | 0.332 | |
| **Smoking Ban at Home (n=140)** ^f^ | |  | |  | |  | |  | |  | |  | |  | |  | |  | |
| No | | 86 (61) | | 34.2 [21.3; 55.0] | | Ref | | 0.34 [0.22; 0.51] | | Ref | | 1.43 [0.95; 2.16] | | Ref | | 21.7 [14.1; 33.2] | | Ref | |
| Yes | | 54 (39) | | 23.6 [13.7; 40.6] | | 0.316 | | 0.34 [0.21; 0.56] | | 0.991 | | 1.33 [0.78; 2.27] | | 0.8328 | | 7.4 [3.84; 14.2] | | **0.004** | |

Abbreviations: GM, geometric mean; CI, confidence interval; Ref, reference group; M, mean; SD, standard deviation; r, Pearson correlation coefficient.  ^a^ n (%) unless otherwise noted. P-values are unadjusted and refer to simple linear regression model results with home and smoking characteristic as the explanatory variable and the contaminant marker in log scale as the response variable. Bold font indicates statistical significance at p < 0.05. ^b^ Based on previous national surveys across the U.S., which indicate that most (69%) homes built before 1960 can have a presence of Pb due to the used of lead-based paint. ^c^ The home size corresponds to the square footage of a home, which was divided based on median size of a single-family home (1500 ft^2^) and the median size of multi-family homes (800 ft^2^) built in the 1960s or earlier in comparison to homes larger than 1500 ft^2^. ^d^ Caregivers who smoked every single day in the last 7 days or not. ^e^ Number of cigarette smokers/day around the child in any location. ^f^ Home Smoking Ban: Smoking is never allowed inside the home.

| **Table 3S. Multivariable regression models of the associations between metal dust loading (*N*=53) and home and smoking characteristics.** | | | | | | | | | | | | |
| --- | --- | --- | --- | --- | --- | --- | --- | --- | --- | --- | --- | --- |
|  | **Lead Loading (µg/m^2^)**  R^2^ = 0.531 (p <0.001) | | | | **Cadmium Loading (µg/m^2^)**  R^2^ = 0.443 (p <0.001) | | | | **Arsenic Loading (µg/m^2^)**  R^2^ = 0.455 (p <0.001) | | | |
| ***Model Fit*** |  |  |  |  |  |  |  |  |  |  |  |  |
|  | $\hat{\boldsymbol{\beta}}$ | **Semi-Partial R^2^** | **95% CI** | **P-value** | $\hat{\boldsymbol{\beta}}$ | **Semi-Partial R^2^** | **95% CI** | **P-value** | $\hat{\boldsymbol{\beta}}$ | **Semi-Partial R^2^** | **95% CI** | **P-value** |
|  |  |  |  |  |  |  |  |  |  |  |  |  |
| **TSNAs Loading (ng/m^2^)** | 0.623 | 0.310 | [0.40; 0.84] | **<0.001** | 0.274 | 0.381 | [0.18; 0.36] | **<0.001** | 0.431 | 0.331 | [0.28; 0.57] | **<0.001** |
| **Housing type** |  |  |  |  |  |  |  |  |  |  |  |  |
| Single-family | Ref |  |  |  | Ref |  |  |  |  |  |  |  |
| Multi-unit apartment | 0.327 | 0.007 | [-0.41; 1.06] | 0.378 | -0.038 | 0.001 | [-0.33; 0.26] | 0.801 | -0.140 | 0.003 | [-0.63; 0.35] | 0.569 |
| **Year House Built** ^a^ |  |  |  |  |  |  |  |  |  |  |  |  |
| <= 1960 | Ref |  |  |  | Ref |  |  |  |  |  |  |  |
| >1960-present | -0.563 | 0.178 | [-1.34; 0.21] | 0.150 | 0.304 | 0.086 | [-0.01; 0.61] | 0.056 | 0.612 | 0.070 | [0.103; 1.14] | **0.019** |
| **House Size** ^b^ | -0.209 | 0.001 | [-0.95; 0.54] | 0.579 | 0.046 | 0.001 | [-0.26; 0.34] | 0.763 | -0.021 | 0.001 | [-0.52; 0.48] | 0.934 |
| **Income** ^c^ |  |  |  |  |  |  |  |  |  |  |  |  |
| ≤ $15,000 | Ref |  |  |  | Ref |  |  |  |  |  |  |  |
| > $15,000 | 0.962 | 0.055 | [0.19; 1.73] | **0.015** | 0.215 | 0.020 | [-0.10; 0.53] | 0.171 | 0.592 | 0.054 | [0.08; 1.10] | **0.025** |
| **Smoking Ban at Home** |  |  |  |  |  |  |  |  |  |  |  |  |
| No | Ref |  |  |  | Ref |  |  |  |  |  |  |  |
| Yes | -0.149 | 0.161 | [-0.97; 0.67] | 0.717 | 0.200 | 0.173 | [-0.25; 0.34] | 0.763 | 0.308 | 0.180 | [-0.24; 0.86] | 0.264 |
| Abbreviations: TSNAs, tobacco-specific-nitrosamines; CI, confidence interval; Ref, reference group. P-value, testing the null hypotheses that the corresponding partial regression coefficient is zero. Bold font indicates statistical significance at p < 0.05 for each parameter. ^a^ Based on previous national surveys across the U.S., which indicate that most (69%) homes built before 1960 can have a presence of Pb due to the use of lead-based paint. ^b^ The home size corresponds to the square footage of the participants’ homes. ^c^ Home Smoking Ban: Smoking is never allowed inside the home. | | | | | | | | | | | | |

| **Table 4S. Multivariable regression model of the association between TSNAs loading and home and smoking characteristics (*N*=53).** | | | | |
| --- | --- | --- | --- | --- |
|  | **TSNAs Loading (ng/m^2^)**  R^2^ = 0.769 (p <0.001) | | | |
| ***Model Fit*** |  |  |  |  |
|  | $\hat{\boldsymbol{\beta}}$ | **Semi-**  **Partial R^2^** | **95% CI** | **P-value** |
|  |  |  |  |  |
| **Nicotine Loading (µg/m^2^)** | 0.826 | 0.478 | [0.66; 0.98] | **<0.001** |
| **Housing type** |  |  |  |  |
| Single-family | Ref |  |  |  |
| Multi-unit apartment | -0.184 | 0.002 | [-0.71; 0.33] | 0.482 |
| **Year House Built** ^a^ |  |  |  |  |
| <= 1960 | Ref |  |  |  |
| >1960-present | -0.533 | 0.141 | [-1.1; 0.01] | 0.052 |
| **House Size** ^b^ | -0.564 | 0.010 | [-1.1; -0.05] | **0.033** |
| **Income** ^c^ |  |  |  |  |
| ≤ $15,000 | Ref |  |  |  |
| > $15,000 | 0.308 | 0.010 | [-0.85; 0.23] | 0.262 |
| **Smoking Ban at Home** |  |  |  |  |
| No | Ref |  |  |  |
| Yes | -0.587 | 0.079 | [-1.1; -0.03] | **0.040** |

| **Table 5S. Descriptive Statistics of Contaminant Dust Concentrations** | | | | | | |  |  | |  |
| --- | --- | --- | --- | --- | --- | --- | --- | --- | --- | --- |
| **Contaminant marker** | **n** | **Mean (SD)** | **GM** | **Min** | **Q1** | **Median** | **Q3** | **Max** | **95% CI** | |
| Dust Lead (µg/g) | 179 | 186.98 (413.4) | 64.36 | 4.31 | 24.98 | 49.91 | 157.78 | 3376.8 | 126.0-147.9 | |
| Dust Arsenic (µg/g) | 179 | 4.37 (5.8) | 3.06 | 0.09 | 2.09 | 3.14 | 4.64 | 51.9 | 3.53-5.23 | |
| Dust Cadmium (µg/g) | 179 | 1.18 (2.1) | 0.73 | 0.03 | 0.42 | 0.76 | 1.20 | 19.8 | 0.87-1.49 | |
| Dust Nicotine (µg/g) | 179 | 63.0 (76.9) | 39.35 | 0.10 | 12.29 | 42.77 | 78.58 | 495.1 | 51.7-74.4 | |
| Dust Total TSNAs (ng/g)* | 158 | 34.5 (57.3) | 7.67 | 0.04 | 4.56 | 18.17 | 35.80 | 458.2 | 25.5-43.5 | |
| *Results include imputed TSNAs values of 0.025 ng/g for the < LOD values. Abbreviations: GM, geometric mean; Q1: first quartile; Q3: third quartile; CI: Confidence Interval  **Ranges of LOQs:** Nicotine (0.001 - 0.011 ng/g or 0.00001 - 0.228 ng/m^2^); individual TSNAs (0.249 - 0.260 ng/g or 0.004 - 5.514 ng/m^2^); Lead (0.410 - 2.742 ng/g or 0.004 - 27.75 ng/m^2^); As and Cd (0.209 – 1.371 ng/g or 0.002 - 28.5 ng/m^2^). | | | | | | | | | | |
